# Medico-Legal

**Published:** 1910-02

**Authors:** John A. Rafter


### PROGRESS IN MEDICAL SCIENCE.

### Medico-Legal

CONDUCTED by JOHN A. RAFTER, M. D.

### SCHOOLS OF PRACTICE,

IN the United States the courts hold all legalised schools or system of medicine as being entirely on a par. If a person announces himself as belonging to any particular system of medicine and practices as such, he cannot be held liable for unfavorable results. The patient who employs such a practitioner and suffers either in limb or life, has no redress under the law. He made his own selection, and must suffer the consequences.

---

### PERTAINING TO FEES.

IT often occurs in case of serious accident, that several physicians are hurriedly called to attend an injured individual, by as many different persons. It is well to know that the third party who so calls the physician, cannot be held legally responsible for his fee, unless there is a specific agreement to that effect. It is a general rule in law that a promise to pay the debt of another to be valid must be in writing. An employer or corporation often calls a physician to attend an injured employee but in the absence of an understanding to that effect no liability is incurred by the employer. The physician must look to the patient for his compensation.

---

### WHAT THE PROSECUTION MUST SHOW.

WHEN the practice of an attending physician or surgeon is called in question in court, the prosecution must show that the alleged injury to the health of the patient was actually caused by the bad treatment of the physician, and also that the bad results could have, and should have, been foreseen and avoided, if ordinary skill and judgment had been used. The courts have held that an error of judgment with no implied malice on the part of the physician is clearly excusable, "if the error was one into which a prudent man might have fallen."

### THERE ARE NO SPECIFICS.

A PHYSICIAN would not be held legally liable if he failed to employ any particular remedy in a given disease, since there are no specific remedies, nor any one remedy upon which all authorities agree, and it is always possible that a sick person might recover without the use of any remedy at all. In cases of poisoning, however, if the physician failed to use the usually accepted antidotes, or in case of a surgical operation, if none of the usually accepted antiseptics, or aseptic methods that are considered essential for favorable results in such cases were used, and if death or serious consequences followed such neglect the physician would be held responsible for criminal carelessness, as he would be considered to have departed entirely from the usually adopted treatment in such cases.

---

### PHYSICIANS' BOOK ACCOUNTS.

IT has been decided by some courts that to enable a physician to recover fees for service by law-suit, that the record for such service should be kept in an intelligent form, and in a regular book; which should contain the original entry. Cabilistic signs not employed in ordinary business transactions and not understood by accountants generally, are not considered sufficient to establish a claim.

---

### WHAT THE DEFENSE MAY DO.

WHEN a physician is called to a person suffering from a wound inflicted by another, and where a surgical operation is necessary in order to save life, the question of legal responsibility becomes a serious one, and the physician may well hesitate. If the patient dies from shock while the operation is being performed or, indeed, if he dies at all the defense is sure to raise the question as to whether death was caused by the wound inflicted or from the operation performed, the anesthetic administered or all combined. In the year 1882, the writer had one of the most disagreeable hour's of his life in attempting to explain to an astute lawyer for the defense that death was not caused by an operation or an anesthetic and that the surgeon was not the actual cause of the individual's death.

---

### ADMINISTERING REMEDIES.

A PHYSICIAN who has a legal right to practise medicine has the right to administer any remedy under the blue canopy that he thinks will benefit his patient; provided that remedy is administered without malice. If however, an unusual or violent remedy is given, and death or serious results ensue, the courts would hold the physician criminally responsible, as the treatment and the results following would imply malice on his part.

---

### CHARGE FOR YOUR SERVICES.

THERE is an old saying, and one that it would be well for physicians to heed, the purport of which is, "never give either salt or advice unless you are asked to do so." Gratuitous advice or service on the part of a physician will not exempt him from an action for malpractice, if either his advice or service has brought about an injury to another. Gratitude for advice of this kind is rather a negligible quality on the part of the lay public as many court records will verify.

---

### RELATIVE TO X-RAY BURNS.

A NUMBER of suits for malpractice brought about by *x*-Ray burns have found their way into the courts during the past few years and have in almost every instance been successfully defended. The same law applies that governs other branches of medical practice. It is to the effect that the operator must have had the ordinary experience, and must possess the ordinary knowledge that others possess doing the same line of work in the community in which he practises. The physician does not guarantee cures, and cannot be held responsible for untoward results if he has used ordinary care and prudence.

---

### A RECENTLY INTRODUCED BILL.

THE leading medical organisations of this state and many prominent members of the bar, have undertaken to have incorporated into the laws of the state a bill to regulate the introduction of medical expert testimony. This same bill was introduced into the assembly in March, 1909, but there was not sufficient time left to give it due consideration and it failed of passage at that session. The bill provides that the justices of the Supreme Court assigned to the Appellate Division in the several departments, should designate a list of physicians and surgeons in each judicial district who may be called as medical expert witnesses by the Trial Court, or by any party to a civil action in any of the courts of this state, and when so called shall testify and be subject to full cross-examinations as other witnesses are. The bill also provides that when called as experts they shall receive for their services and attendance such sums as the presiding judge may allow to be paid at once by the treasurer, or other fiscal officer of the county in which the trial is held. It is stated in the bill that nothing in it shall be construed as limiting the right of parties to call other expert witnesses as heretofore. The proposed measure is intended only to regulate testimony in criminal, not in civil suits.—*Journal of the A. M. A.*, January 15, 1910.

